# Axillary surgery in patients with breast cancer with one to three positive micro- or macrometastases in the sentinel lymph nodes: an observational study

**DOI:** 10.1007/s12282-025-01726-2

**Published:** 2025-05-25

**Authors:** Shigeru Imoto, Hiroyuki Yasojima, Takeshi Nagashima, Tatsuya Onishi, Tsutomu Takashima, Masahiro Kitada, Masaya Kawada, Tetsu Hayashida, Yasuto Naoi, Tomohiko Aihara, Noriaki Wada, Hidetaka Kawabata, Masayuki Yoshida, Uhi Toh, Kimiyasu Yoneyama, Akimitsu Yamada, Hitoshi Tsuda, Norikazu Masuda, Mari Saito-Oba, Junichi Sakamoto

**Affiliations:** 1 Department of Breast Surgery, Kyorin University Hospital, 6-20-2 Shinkawa, Mitaka, Tokyo 181-8611 Japan; 2 NHO Osaka National Hospital, Osaka, Japan; 3 Chiba University Hospital, Chiba, Japan; 4 National Cancer Center Hospital East, Kashiwa, Japan; 5 Nara Prefecture Seiwa Medical Center, Ikoma, Japan; 6 Asahikawa Medical University Hospital, Asahikawa, Japan; 7 Tonan Hospital, Sapporo, Japan; 8 Keio University School of Medicine, Tokyo, Japan; 9 Kyoto Prefectural University of Medicine, Kyoto, Japan; 10 Aihara Hospital, Minoh, Japan; 11 Tokyo Dental College Ichikawa General Hospital, Ichikawa, Japan; 12 Toranomon Hospital, Tokyo, Japan; 13 Seirei Social Welfare Community, Seirei Center for Health Promotion and Preventive Medicine, Hamamatsu, Japan; 14 Kurume University Hospital, Kurume, Japan; 15 Hiratsuka City Hospital, Hiratsuka, Japan; 16 Yokohama City University Hospital, Yokohama, Japan; 17 Chiba Medical Center, Chiba, Japan; 18 Graduate School of Medicine, Kyoto University, Kyoto, Japan; 19 National Center of Neurology and Psychiatry, Kodaira, Japan; 20 Tokai Central Hospital, Kakamigahara, Japan

**Keywords:** Breast cancer, Sentinel node biopsy, Regional nodal irradiation, Regional node recurrence

## Abstract

**Background:**

The Japanese Society for Sentinel Node Navigation Surgery conducted a multi-institutional prospective cohort study to compare sentinel node biopsy (SNB) with SNB followed by axillary lymph node dissection (ALND) in breast cancer patients with positive sentinel lymph node (SLN).

**Patients and methods:**

Female patients with breast cancer with cT1-3N0-1M0 were eligible. In cases of one to three positive micro- or macrometastases in the SLN confirmed by histological or molecular diagnosis, SNB alone (SNB group) or additional ALND (ALND group) was performed under physician’s discretion. The primary endpoint was the 5-year regional node (RN) recurrence rate in the SNB group. Propensity score matching (PSM) was performed to compare the prognosis between the two groups.

**Results:**

Of the 871 eligible cases registered between 2013 and 2016, 308 underwent SNB alone. At the median follow-up of 6.3 years, 5-year RN recurrence rate was 2.7%. After PSM, 209 cases were matched in each group. Breast-conserving surgery and mastectomy were performed in 225 (54%) and 193 cases (46%), respectively. One-positive SLN was recorded in 366 cases (88%), two in 48 (11%), and three in 4 (1%). Macro- and micrometastases in SLN were diagnosed in 271 (65%) and 147 cases (35%), respectively. Regional nodal irradiation (RNI) was performed in 42 (20%) and 13 (6%) cases of the SNB and ALND group. The 5-year RN recurrence rate was 2.1% and 2.0%, respectively.

**Conclusions:**

ALND is not necessary for early breast cancer patients with one-positive SLN despite type of breast surgery.

**Supplementary Information:**

The online version contains supplementary material available at 10.1007/s12282-025-01726-2.

## Introduction

According to the results of ACOSOG Z0011, IBCSG 23-01, and AMAROS trials, axillary surgery in pathologically node-positive breast cancer tends to be less invasive with sentinel node biopsy (SNB) followed by adjuvant therapy and/or regional nodal irradiation (RNI) instead of axillary lymph node dissection (ALND) [1–3]. The recurrence rates of regional node (RN) at 10 years after no ALND were 1.5%, 2%, and 1.8% in the three studies. The rates were extremely low, but additional ALND revealed 13% to 33% of nodal metastases outside the sentinel lymph node (SLN). The recent ASCO guideline regarding axillary management in early breast cancer recommended RNI for regional control in SLN-positive breast cancer [4]. However, necessity for RNI in case of fewer nodal metastases is still debatable [5]. Theoretically, patients with breast cancer with only SLN involvement have no clinical benefit from RNI. The Japanese Society for Sentinel Node Navigation Surgery (SNNS) was founded in 1999 to advance basic and clinical research regarding sentinel node concept in solid tumors. Thus, we conducted a multi-institutional prospective cohort study to compare SNB with SNB followed by ALND in SLN-positive breast cancer (UMIN No. 000011782). The study complied with the Declaration of Helsinki and the Ethical Guidelines for Clinical Research of the Ministry of Health, Labor, and Welfare of Japan. All the patients provided written informed consent before participation. The study was approved by the institutional review board at Kyorin University School of Medicine.

## Patients and methods

Previously, we reported the trial note of this observational study [6]. In brief, female patients with breast cancer with cT1-3 N0-1M0 were eligible. In cases of one to three positive micro- or macrometastases in the SLN confirmed by histological or molecular diagnosis, SNB alone or SNB followed by ALND was performed under physician’s discretion in the operation theatre. Primary chemotherapy before or after SNB was acceptable for registration. Lymph node sampling was also accepted in the SNB group. Cases of bilateral breast cancer, isolated tumor cells only in the SLN, and medical history of invasive cancer within 5 years at the time of registration were ineligible. The primary endpoint was the 5 year RN recurrence rate in the SNB group. The secondary endpoint was the 5-year overall survival (OS) rate. We planned to recruit 240 patients treated with SNB to reject that the 5-year RN recurrence rate was more than 10% assuming the rate 5%. To demonstrate the de-escalation of axillary surgery through comparison of the SNB and ALND groups, propensity score matching (PSM) was performed. Matching variables were selected as clinicopathological factors before decision-making of axillary surgery: initial treatment of breast cancer, method of breast surgery, metastatic tumor size and number of SLN, clinical stage, age (50 years or younger, 51 to 70 years, 71 years or older), body mass index (less than 18.5, 18.5 to 25, 25 or more), menopausal status, family history of breast cancer and medical history of invasive disease. Patients were matched on their propensity scores using a 1:1 greedy matching technique. RN recurrence-free survival, recurrence-free survival (RFS) and OS were analyzed using the Kaplan–Meier method. Statistical significance was set at p-value < 0.05. Statistical analysis was performed using SAS 9.4 (SAS Institute, Cary, NC).

## Results

**Fig. 1:**
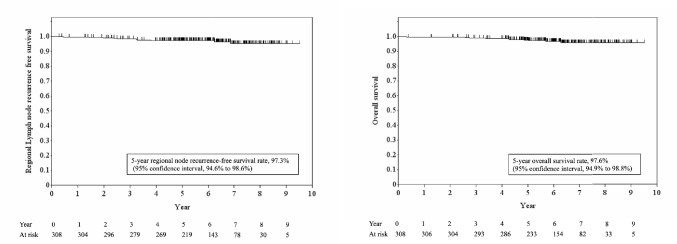
Regional node recurrence-free survival (left) and overall survival (right) in 308 cases of the SNB group

A total of 883 cases were registered from 26 participating institutes between 2013 and 2016. After exclusion of 12 ineligible cases, 871 cases were examined for statistical analysis. Among them, 308 cases belonged to the SNB group in which 43 cases (14%) underwent SNB with lymph node sampling (Table 1). SLN metastasis was diagnosed by hematoxylin–eosin staining, while one-step nucleic acid amplification assay was attempted in 30 cases (3%) [7]. The number of SLNs ranged from 1 to 11 (median, 2.5; mean, 2.5). When compared between the SNB and ALND groups using the chi-squared test, the SNB group had significantly higher proportion of patients with early stage, invasive ductal carcinoma, lower nuclear grade, micrometastatic SLN, breast-conserving surgery followed by radiation therapy, radioisotope-guided or indocyanine green (ICG)-fluorescence-guided lymphatic mapping, fewer numbers of SLN metastases, no chemotherapy, and axillary nodal irradiation. It was statistically different in the numbers of SLNs examined in the SNB and ALND group (p = 0.01 at non-paired t-test). At the median follow-up of 6.3 years, 5-year RN recurrence rate was 2.7% (95% confidence interval [CI] 1.4–5.4%) and 5 year OS rate was 97.6% (94.9–98.8%) (Fig. 1).

**Table 1:** Patient background for eligible cases

		ALND group	SNB group	Total	P value at Chi-squared test
No. of cases (%)		563 (100)	308 (100)	871 (100)	
Age (y.o.)	Mean [median]	54.9 [52]	55.6 [53]		
	50 or less	228 (40.5)	111 (36)	339 (38.9)	0.409
	51 to 70	268 (47.6)	160 (51.9)	428 (49.1)	
	71 or more	67 (11.9)	37 (12)	104 (11.9)	
Body mass index (kg/m2)	Mean [median]	22.6 [22.1]	22.8 [22.2]		
	< 18.5	63 (11.2)	26 (8.4)	89 (10.2)	0.440
	18.5- < 25	377 (67)	212 (68.8)	589 (67.6)	
	25-	123 (21.8)	70 (22.7)	193 (22.2)	
Menopausal status	Pre-	274 (48.7)	145 (47.1)	419 (48.1)	0.653
	Post-	289 (51.3)	163 (52.9)	452 (51.9)	
Family history of breast cancer	No	503 (89.3)	274 (89)	777 (89.2)	0.862
	Yes	60 (10.7)	34 (11)	94 (10.8)	
History of invasive cancer	No	548 (97.3)	304 (98.7)	852 (97.8)	0.187
	Yes	15 (2.7)	4 (1.3)	19 (2.2)	
Clinical stage	I	228 (40.5)	148 (48.1)	376 (43.2)	0.031
	IIA	275 (48.8)	138 (44.8)	413 (47.4)	
	IIB	56 (9.9)	20 (6.5)	76 (8.7)	
	IIIA	4 (0.7)	2 (0.6)	6 (0.7)	
Histology	IDC	504 (89.5)	283 (91.9)	777 (89.2)	0.046
	ILC	36 (2.3)	13 (1.3)	17 (1.9)	
	Others	17 (5.5)	9 (7.5)	54 (6.2)	
	Unknown	6 (1.1)	3 (0.9)	9 (1.0)	
Nuclear grade	1	193 (34.3)	101 (32.8)	294 (33.8)	0.050
	2	229 (40.7)	139 (45.1)	368 (42.3)	
	3	113 (20.1)	52 (16.9)	165 (18.9)	
	Unknown	28 (5.0)	16 (5.2)	44 (5.1)	
Tumor subtype	Luminal	505 (89.7)	272 (88.3)	777 (89.2)	0.053
	HER2	13 (2.3)	4 (1.3)	17 (1.9)	
	Triple-negative	31 (5.5)	23 (7.5)	54 (6.2)	
	Unknown	14 (2.5)	9 (2.9)	23 (2.6)	
Primary therapy	Surgery	475 (84.4)	242 (78.6)	717 (82.3)	0.0002
	SNB prior to neoadjuvant therapy	28 (5)	39 (12.7)	67 (7.7)	
	Neoadjuvant therapy	60 (10.7)	27 (8.8)	87 (10.0)	
SNB with lymph node sampling	Yes	0 (0)	43 (14.0)	43 (4.9)	
Breast surgery	Breast-conserving surgery	264 (46.9)	172 (55.8)	436 (50.1)	0.012
	Mastectomy	299 (53.1)	136 (44.2)	435 (49.9)	
Lymphatic mapping	Dye-guided	537 (95.4)	279 (90.6)	816 (93.7)	0.003
	Radioisotope-guided	309 (54.9)	216 (70.1)	525 (60.3)	
	ICG-fluorescence-guided	45 (8.4)	45 (14.6)	90 (10.3)	
	CT-guided	41 (7.3)	18 (5.8)	59 (6.8)	
No. of SLNs examined	Mean (Median)	2.4 (2.0)	2.7 (2.0)	2.5 (2.5)	0.010*
	Range	1–11	1—7	1—11	
No. of SLN metastases	1	410 (72.8)	272 (88.3)	682 (78.3)	< 0.0001
	2	126 (22.4)	34 (11)	160 (18.4)	
	3	27 (4.8)	2 (0.6)	29 (3.3)	
Metastatic tumor size in SLN	Macrometastasis	488 (86.7)	142 (46.1)	630 (72.3)	< 0.0001
	Micrometastasis	75 (13.3)	166 (53.9)	241 (27.7)	
Chemotherapy	Neoadjuvant	34 (6.0)	17 (5.5)	51 (5.9)	0.001
	Adjuvant	228 (40.5)	86 (27.9)	314 (36.1)	
	No	301 (53.5)	205 (66.6)	506 (58.1)	
Endocrine therapy	Neoadjuvant	39 (6.9)	29 (9.4)	68 (7.8)	0.100
	Adjuvant	475 (84.4)	242 (80.2)	717 8.2)	
	No	49 (8.7)	37 (12.0)	84 (9.6)	
Radiation therapy	Breast	248 (44.0)	162 (52.6)	410 (47.1)	< 0.0001
	Chest wall	54 (9.6)	17 (5.5)	71 (8.2)	
Regional nodal irradiation	Yes	84 (14.9)	57 (18.5)	141 (16.2)	0.170
	No	479 (85.1)	251 (81.5)	730 (83.8)	
Radiation field of regional node	Axillary LN	18 (3.2)	47 (15.6)	65 (7.5)	< 0.0001
	Supraclavicular LN	79 (14.0)	14 (4.5)	93 (10.7)	
	Parasternal LN	9 (1.6)	3 (1.0)	12 (1.4)	

*ALND* axillary lymph node dissection, *ICG* indocyanine green, *IDC* invasive ductal carcinoma, *ILD* invasive lobular carcinoma, *LN* lymph nodes, *SLN* sentinel lymph nodes, *SNB* sentinel node biopsy

P value marked with an asterisk was calculated by using non-paired t test

**Fig. 2:**
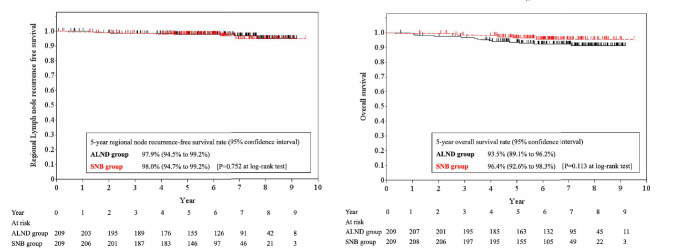
Regional node recurrence-free survival (left) and overall survival (right) in the SNB and ALND group after propensity score matching

**Fig. 3:**
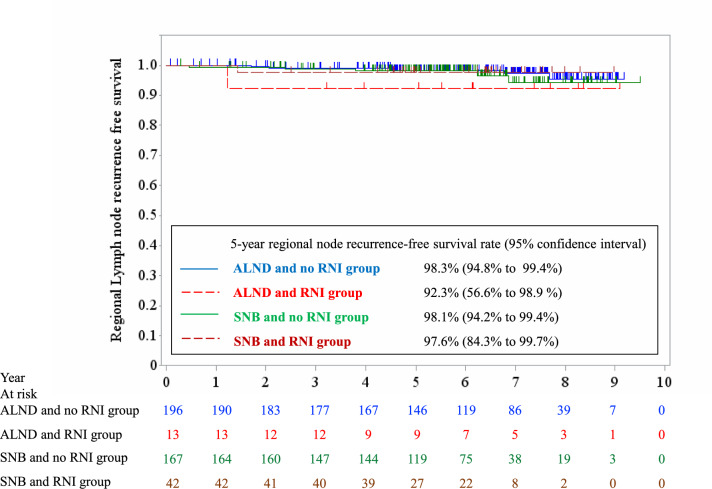
Regional node recurrence-free survival in the SNB and ALND group treated with or without regional nodal irradiation (RNI) after propensity score matching

After PSM, 209 cases were matched in each group (Table 2). Among them, 343 cases (82%) received surgery first. Breast-conserving surgery and total mastectomy was performed in 225 (54%) and 193 cases (46%), respectively. One-positive SLN was recorded in 366 cases (88%), two in 48 (11%), and three in 4 (1%). Macro- and micrometastases in SLN were diagnosed in 271 (65%) and 147 cases (35%), respectively. Three-hundred seventy-six cases (90%) had luminal-like subtype. RNI was statistically more frequently performed in the SNB group, but RNI was planned only in 42 cases (20%) of the SNB group and 13 cases (6%) of the ALND group. The 5-year RN recurrence rate was 2.0% (95% CI 0.8–5.3%) and 2.1% (0.8– 5.5%) for the SNB and ALND group, respectively (Hazard ratio [HR] = 1.20; 95% CI 0.38–3.78, p = 0.752 at log-rank test) (Fig. 2). When RN recurrence in the SNB group after PSM was observed according to RNI, the 5-year RN recurrence rate was 1.9% (0.6–5.8%) in 167 cases without RNI and 2.4% (0.3–15.7%) in 42 cases with RNI (Fig. 3). The 5-year OS rate was 96.4% (92.6–98.3%) and 93.5% (89.1–96.2%) for the SNB and ALND group, respectively (HR = 0.51; 95% CI 0.22–1.19, p = 0.113) (Fig. 2). We also examined RFS. Recurrence and death in eligible and matched cases are summarized in Table 3. The 5-year RFS rate in the eligible cases was 92.9% (89.3–95.3%) and 88.1% (85.1–90.6%) for the 2 groups, respectively. After PSM, it was 92.6% (88.0–95.5%) and 88.7% (83.5–92.4%) for the 2 groups, respectively (HR = 0.70; 95% CI 0.39–1.25, p = 0.225).

**Table 2:** Patient background for the propensity score matching

		ALND group	SNB group	Total	P value at chi-squared test
No. of cases (%)		209 (100)	209 (100)	418 (100)	
Age (y.o.)	50 or less	77 (36.8)	80 (38.3)	157 (37.6)	0.952
	51 to 70	112 (53.6)	109 (52.2)	221 (52.9)	
	71 or more	20 (9.6)	20 (9.6)	40 (9.6)	
Body mass index (kg/m^2^)	< 18.5	23 (11.0)	21 (10.1)	44 (10.5)	0.935
	18.5- < 25	141 (67.5)	141 (67.5)	282 (67.5)	
	25-	45 (21.5)	47 (22.5)	92 (22.0)	
Menopausal status	Pre-	105 (50.2)	104 (49.8)	209 (50.0)	0.922
	Post-	104 (49.8)	105 (50.2)	209 (50.0)	
Family history of breast cancer	No	190 (90.9)	185 (88.5)	375 (89.7)	0.421
	Yes	19 (9.1)	24 (11.5)	43 (9.6)	
History of invasive cancer	No	206 (98.6)	205 (98.1)	411 (98.3)	0.703
	Yes	3 (1.4)	4 (1.9)	7 (1.7)	
Clinical stage	I	96 (45.9)	98 (46.9)	194 (46.4)	0.845
	IIA, IIB, IIIA	113 (54.1)	111 (53.1)	224 (53.6)	
Histology	IDC	186 (89.0)	187 (89.5)	373	0.682
	ILC	12 (5.7)	11 (5.3)	23 (55.0)	
	Others	9 (4.3)	7 (3.3)	16 (3.8)	
	Unknown	2 (1.0)	4 (1.9)	6 (1.4)	
Nuclear grade	1	67 (32.1)	65 (31.1)	132 (31.6)	0.260
	2	86 (41.1)	103 (49.3)	189 (45.2)	
	3	48 (23.0)	34 (16.3)	82 (19.6)	
	Unknown	8 (3.8)	7 (3.3)	15 (3.5)	
Tumor subtype	Luminal	184 (88.0)	192 (91.9)	376 (89.9)	0.440
	HER2	2 (1.0)	3 (1.4)	5 (1.2)	
	Triple-negative	15 (7.2)	10 (4.8)	25 (6.0)	
	Unknown	8 (3.8)	4 (1.9)	12 (2.9)	
Primary therapy	Surgery	174 (83.3)	169 (80.9)	343 (82.1)	0.810
	SNB prior to neoadjuvant therapy	18 (8.6)	20 (9.69)	38 (9.1)	
	Neoadjuvant therapy	17 (8.1)	20 (9.6)	37 (8.9)	
SNB with lymph node sampling	Yes	0 (0)	32 (15.3)	32 (7.7)	
Breast surgery	Breast-conserving surgery	108 (51.7)	117 (56.0)	225 (53.8)	0.377
	Mastectomy	101 (48.3)	92 (44.0)	193 (46.2)	
Lymphatic mapping	Dye-guided	194 (92.8)	190 (90.1)	384 (91.9)	0.105
	Radioisotope-guided	117 (56.0)	144 (68.9)	261 (62.4)	
	ICG-fluorescence-guided	16 (7.7)	32 (15.3)	48 (11.5)	
	CT-guided	9 (4.3)	12 (5.7)	21 (5.0)	
No. of SLNs examined	Mean (Median)	2.3 (2.0)	2.7 (3.0)	2.5 (2.4)	0.003*
	Range	1–11	1—7	1—11	
No. of SLN metastases	1	187 (89.5)	179 (85.7)	366 (87.6)	0.470
	2	20 (9.6)	28 (13.4)	48 (11.4)	
	3	2 (1.0)	2 (1.0)	4 (1.0)	
Metastatic tumor size in SLN	Macrometastasis	136 (65.1)	135 (64.6)	271 (64.8)	0.918
	Micrometastasis	73 (34.9)	74 (35.4)	147 (35.2)	
Chemotherapy	Neoadjuvant	20 (9.6)	25 (12.0)	45 (10.8)	0.724
	Adjuvant	86 (41.1)	82 (39.2)	168 (40.2)	
	No	120 (57.4)	121 (57.9)	241 (57.7)	
Endocrine therapy	Neoadjuvant	16 (7.7)	18 (8.6)	34 (8.1)	0.850
	Adjuvant	184 (88.0)	187 (89.5)	371 (88.8)	
	No	25 (12.0)	22 (10.5)	47 (11.2)	
Radiation therapy	Breast	98 (46.9)	110 (52.6)	208 (49.8)	0.260
	Chest wall	6 (2.9)	12 (5.7)	18 (4.3)	
Regional nodal irradiation	Yes	13 (6.2)	42 (20.1)	55 (13.2)	< 0.0001
	No	196 (93.8)	167 (79.9)	363 (86.8)	
Radiation field of regional node	Axillary LN	5 (2.4)	34 (16.3)	39 (9.3)	0.002
	Supraclavicular LN	11 (5.3)	10 (4.8)	21 (5.0)	
	Parasternal LN	0 (0)	3 (1.4)	3 (0.7)	

*ALND* axillary lymph node dissection, *ICG* indocyanine green, *IDC* invasive ductal carcinoma, *ILD* invasive lobular carcinoma, *LN* lymph nodes, *SLN* sentinel lymph nodes, *SNB* sentinel node biopsy

P value marked with an asterisk was calculated by using non-paired t test

**Table 3:** Recurrence and death for eligible cases and marched cases

	Eligible cases		Matched cases	
	ALND group	SNB group	ALND group	SNB group
No. of cases	563	308	209	209
Variable				
Recurrence-no. (%)	79 (14.0)	28 (9.1)	29 (13.9)	19 (9.1)
Local	24 (4.3)	6 (1.9)	10 (4.8)	3 (1.4)
Regional	23 (4.1)	11 (3.6)	9 (4.3)	7 (3.4)
Distant	56 (10)	19 (6.1)	20 (9.6)	13 (6.2)
Death-no. (%)	35 (6.2)	10 (3.3)	16 (7.7)	8 (3.8)
Cause of death-no./total no. (%)				
Breast cancer	28 (80)	5 (50)	14 (87.5)	4 (50)
Other cancer	2 (5.7)	2 (20)	1 (6.3)	2 (25)
Other disease	5 (14.3)	3 (30)	1 (6.3)	2 (25)

*ALND* axillary lymph node dissection, *SNB* sentinel node biopsy

In this study, over 70% of eligible cases had only one-positive SLNs. On the other hand, optimized treatment after SNB only in cases with 2 or 3-positive SLNs should be discussed carefully. We additionally demonstrated the 5 year RN recurrence rates in the SNB and ALND groups after PSM according to the positive numbers of SLNs (1 vs. 2 or 3) (Supplementary Fig. 1). According to the matched cases with 2 or 3-positive SLNs, the 5-year RN recurrence-free rate for the SNB and ALND group was 96.3% (76.5–99.5%) and 100% (95% CI 100–100%), respectively (p = 0.15 at log-rank test). Although RN recurrence curves were similar among the 4 groups, late recurrence of RN was observed in the SNB group with 2 or 3 positive SLNs. It is suggested that SNB only should be carefully considered in multiple SLNs-positive case.

## Discussion

From a prospective cohort study, de-escalation surgery in the axilla was feasible in SLN-positive breast cancer. Table 4 shows the comparison of RN recurrence among our study (SNNS), ACOSOG Z0011 and AMAROS trials [3, 8]. The 5-year RN recurrence rate after no ALND was 2.1%, 1.5%, and 1.2%, respectively. Although eligible case and protocol design were different in those studies, regional control after SNB alone was acceptable. ACOSOG Z0011 reported that breast irradiation in some cases had been planned with high tangent field or posterior axillary boost [9]. RNI may be effective for eradication of occult disease in the axilla. However, in our study, 20% of cases in the SNB group received RNI. One reason is that most cases had one positive-SLN and participating physicians did not consider RNI necessary. Another reason is that most cases had luminal-like subtype and adjuvant endocrine therapy was considered as control for occult disease. According to NSABP B32 trial, 15.9% of 3887 patients with negative SLN revealed occult metastases in lymph node specimens sectioned deeper from the tissue block [10]. A multivariate analysis demonstrated that occult metastasis was an independent poor prognostic factor of distant disease and death. However, endocrine therapy had favorable hazard ratios for both events. In our study, 90% of cases for PSM had luminal-like subtype and 371 cases (89%) received some kinds of adjuvant endocrine therapy (Table 2). In addition, 88% of cases for PSM had one-positive SLN. When considered with similar outcomes for the SNB and ALND groups for PSM, occult disease in luminal-like cases may be controlled with adjuvant endocrine therapy regardless of RNI. The 5-year RFS and OS were not statistically different in the SNB and ALND groups. Although we used the Kaplan–Meier method, competing risks such as distant recurrence or death may influence RN recurrence and should be considered in future studies.

**Table 4:** Comparison of regional nodal recurrence among SNNS, ACOSOG Z0011 and AMAROS trials

		SNNS cases for PSM		ACOSOG Z0011 Follow-up cases		AMAROS	
		ALND group	SNB group	ALND group	SNB group	ALND group	Axillary radiotherapy group
No. of cases (%)		209 (100)	209 (100)	388 (100)	425 (100)	744 (100)	681 (100)
Breast surgery	Partial mastectomy	108 (52)	117 (56)	388 (100)	425 (100)	609 (82)	557 (82)
	Total mastectomy	101 (48)	92 (44)	0 (0)	0 (0)	127 (17)	121 (18)
Regional nodal irradiation	Yes	13 (6)	42 (20)	Out of protocol		41 (6)	681 (100)
No. of SLN metastases	1	187 (89)	179 (86)	199 (58)	295 (71)	581 (78)	512 (75)
	2	20 (10)	28 (13)	68 (20)	76 (18)	127 (17)	134 (20)
	3 or more	2 (1)	2 (1)	72 (21)	15 (4)	36 (5)	35 (5)
Metastatic tumor size in SLN	Macrometastasis	136 (65)	135 (65)	228 (63)	202 (55)	442 (59)	419 (62)
	Micrometastasis	73 (35)	74 (35)	137 (37)	164 (45)	215 (29)	195 (29)
	Isolated tumor cells	0 (0)	0 (0)	0 (0)	0 (0)	87 (12)	67 (10)
Regional node recurrence rate	At 5 year’s follow-up	2.0%	2.1%	0.5%	1.5%	0.4%	1.2%

*ACOSOG* American college of surgeons oncology group, *ALND* axillary lymph node dissection, *AMAROS* after mapping of the axilla: radiotherapy or surgery, *PSM* propensity score matching, *SLN* sentinel lymph nodes, *SNB* sentinel node biopsy

We did not include the number of SLNs on a matching valuable for PSM, because mapping technique for different tracers influenced the number of SLNs [11]. However, it was statistically different in the numbers of SLNs examined between the SNB and ALND group before and after PSM (Tables 1, 2). The difference at the mean number was very small (0.3 before PSM and 0.4 after PSM), but total numbers of SLNs might influence decision-making of axillary surgery at operating theater.

Few randomized trials have compared SNB with ALND for patients with SLN-positive breast cancer who underwent mastectomy. The National Cancer Database in 2018 reported that ALND was performed in 46% of one to two SLN-positive cases treated with mastectomy [12]. From a multivariate analysis, age < 50 years, cT2, two-positive SLNs, and Hispanic ethnicity were independently associated with patients undergoing ALND. In our study, participating physicians also preferred to ALND in cases treated with mastectomy (Table 1). However, RN recurrence rates were similar in the SNB and ALND groups regardless of breast surgery after PSM. According to RNI in SLN-positive breast cancer, M. D. Anderson Cancer Center reported that the 5-year RN recurrence rate was only 1.2% in patients treated with SNB alone without RNI in a contemporary cohort [13]. SINODAR-ONE trial is a prospective, noninferiority, multicenter, randomized study to assess the therapeutic role of ALND for patients with T1-2 breast cancer with one or two-macrometastatic SLNs. At the median follow-up of 34 months in 879 cases of intention-to treat population, the 3-year survival and relapse rates of patients treated with SNB alone were not inferior to those of patients treated with ALND. RN recurrence in the axilla was observed in each one patient treated with SNB and ALND [14]. According to a subset analysis of 218 patients treated with mastectomy, the 5-year OS and recurrence-free survival rates were similar among 107 patients treated with SNB alone and 111 patients treated with ALND [15]. To validate omitting ALND in breast cancer with 1 or 2 macrometastatic SLNs, a large phase III study, SENOMAC trial, reported non-inferiority to SNB alone regarding the prespecified secondary end point of recurrence-free survival [16]. The estimated 5-year recurrence-free survival was 89.7% (95% CI 87.5–91.9%) in the SNB group and 88.7% (95% CI 86.3–91.1%) in the ALND group. In this study, RN recurrence rate was 0.4% and 0.5%, respectively. On the other hand, approximately one-third of the patients had extracapsular extension in the SLN and most patients in the SNB group received adjuvant therapy and RNI. It is well known that extracapsular extension is a strong predictive factor of non-SLN metastasis [17]. Since necessity of RNI remains undetermined in breast cancer patients with low-risk of non-SLN metastasis, optimal treatment of the axilla in patients with SLN-positive breast cancer will be clarified in POSNOC trial [5]. The primary endpoint is to prove that adjuvant therapy alone is not inferior to adjuvant therapy plus axillary treatment with ALND or RNI in female breast cancer with 1 or 2 macrometastatic SLNs.

Adverse events (AE) and quality of life (QoL) are the important issues of axillary surgery in breast cancer. ALMANAC trial first reported arm morbidity and QoL for the primary endpoints after SNB and ALND in cases of clinically node-negative breast cancer [18]. The absolute rate of arm lymphedema at 12 months after SNB and ALND was statistically different (5% and 13%, respectively). Overall patient-reported QoL and arm functioning scores were also statistically better in the SNB group. SENOMAC trial reported patient-reported outcomes at 1 year after axillary surgery [19]. From the results, participants receiving SNB only had significantly lower symptom scores on the EORTC subscales of pain, arm symptoms and breast symptoms. Physical function, mental function and mobility activities were also significantly in favor of the SNB group. Unfortunately, we had no planning to measure AE and QoL in enrolled patients [6] and the lack of these evaluations represents a limitation of our study. In POSNOC trial, arm morbidity, QoL, anxiety and economic outcomes will be examined as secondary endpoints among patients who received SNB and adjuvant therapy with or without additional axillary treatment.

In conclusion, ALND is not necessary for early breast cancer patients with one-positive SLN despite type of breast surgery. RNI after SNB alone in such cases should be carefully discussed.

## Supplementary Information

Below is the link to the electronic supplementary material.

Supplementary file1 (PPTX 186 KB)

## Data Availability

The datasets generated and analyzed in this study are not publicly available due to protocol policy by SNNS.
