# Supplementary material for: Axillary surgery in patients with breast cancer with one to three positive micro- or macrometastases in the sentinel lymph nodes: an observational study
Source: Breast Cancer. 2025 May 25;32(5):1013–22. doi: 10.1007/s12282-025-01726-2 (PMC12394296; doi:10.1007/s12282-025-01726-2)
Supplement: Supplementary file 1 — Supplementary file1 (PPTX 186 KB) [file 12282_2025_1726_MOESM1_ESM.pptx]

## Slide 1
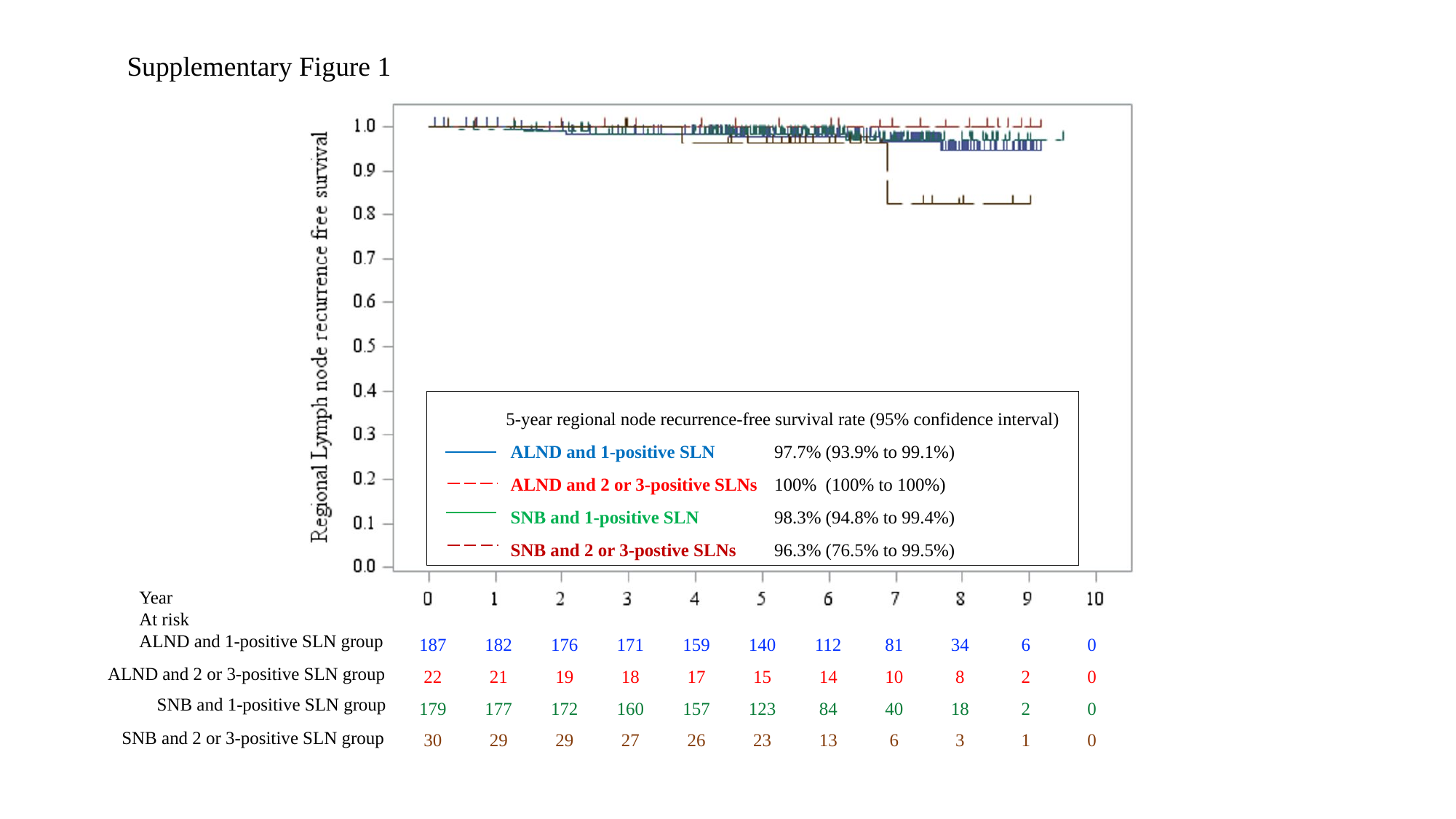

Supplementary Figure 1
 5-year regional node recurrence-free survival rate (95% confidence interval)
 ALND and 1-positive SLN	 97.7% (93.9% to 99.1%)
 ALND and 2 or 3-positive SLNs	 100% (100% to 100%)
 SNB and 1-positive SLN	 98.3% (94.8% to 99.4%)
 SNB and 2 or 3-postive SLNs	 96.3% (76.5% to 99.5%)
Year
At risk
ALND and 1-positive SLN group
| 187 | 182 | 176 | 171 | 159 | 140 | 112 | 81 | 34 | 6 | 0 |
| --- | --- | --- | --- | --- | --- | --- | --- | --- | --- | --- |
| 22 | 21 | 19 | 18 | 17 | 15 | 14 | 10 | 8 | 2 | 0 |
| 179 | 177 | 172 | 160 | 157 | 123 | 84 | 40 | 18 | 2 | 0 |
| 30 | 29 | 29 | 27 | 26 | 23 | 13 | 6 | 3 | 1 | 0 |
ALND and 2 or 3-positive SLN group
SNB and 1-positive SLN group
SNB and 2 or 3-positive SLN group
